# Supplementary material for: Correlation of RECIST, Computed Tomography Morphological Response, and Pathological Regression in Hepatic Metastasis Secondary to Colorectal Cancer: The AVAMET Study
Source: Cancers (Basel). 2020 Aug 12;12(8):2259. doi: 10.3390/cancers12082259 (PMC7465835; doi:10.3390/cancers12082259)
Supplement: Supplementary file 1 [file cancers-12-02259-s001.pdf]

Supplementary material

Correlation of RECIST, Computed Tomography Morphological Response, and Pathological Regression in Hepatic Metastasis Secondary to Colorectal Cancer: The AVAMET Study

Ruth Vera, María Luisa Gómez, Juan Ramón Ayuso, Joan Figueras, Pilar García-Alfonso, Virginia Martínez, Adelaida Lacasta, Ana Ruiz-Casado, María José Safont, Jorge Aparicio, Juan Manuel Campos, Juan Carlos Cámara, Marta Martín-Richard, Clara Montagut, Carles Pericay, Jose María Vieitez, Esther Falcó, Mónica Jorge, Miguel Marín, Mercedes Salgado and Antonio Viúdez

Table S1. Adverse events of interest with bevacizumab (safety population; *n* = 78).

Adverse Event, *n* (%)	All grades	Grade 1/2	Grade 3/4
Oropharyngeal hemorrhage	6 (8)	5 (6)	1 (1)
Hypertension	5 (6)	4 (5)	1 (1)
Rectal hemorrhage	5 (6)	5 (6)	0
Proteinuria	4 (5)	4 (5)	0
Pulmonary embolism	4 (5)	1 (1)	3 (4)
Post-procedural hemorrhage	2 (3)	1 (1)	1 (1)
Hematuria	1 (1)	1 (1)	0
Wound evisceration	1 (1)	0	1 (1)
Cerebrovascular accident	1 (1)	0	1 (1)
Anal fissure	1 (1)	1 (1)	0
Anastomotic fistula	1 (1)	0	0
Jejunal perforation	1 (1)	0	1 (1)
Hemoptysis	1 (1)	1 (1)	0
Bleeding in the stomal area	1 (1)	1 (1)	0

© 2020 by the authors. Licensee MDPI, Basel, Switzerland. This article is an open access article distributed under the terms and conditions of the Creative Commons Attribution (CC BY) license (http://creativecommons.org/licenses/by/4.0/).
